# The role of the Hippo pathway in human disease and tumorigenesis

**DOI:** 10.1186/2001-1326-3-25

**Published:** 2014-07-18

**Authors:** Daniel A Barron, Jacob D Kagey

**Affiliations:** 1 Department of Cell Biology, Emory University School of Medicine, Atlanta, GA, USA; 2 Department of Biology, University of Detroit Mercy, 4001 West McNichols Road, Detroit, MI, USA

**Keywords:** Cancer, Hippo signaling, YAP, Apoptosis

## Abstract

Understanding the molecular nature of human cancer is essential to the development of effective and personalized therapies. Several different molecular signal transduction pathways drive tumorigenesis when deregulated and respond to different types of therapeutic interventions. The Hippo signaling pathway has been demonstrated to play a central role in the regulation of tissue and organ size during development. The deregulation of Hippo signaling leads to a concurrent combination of uncontrolled cellular proliferation and inhibition of apoptosis, two key hallmarks in cancer development. The molecular nature of this pathway was first uncovered in *Drosophila melanogaster* through genetic screens to identify regulators of cell growth and cell division. The pathway is strongly conserved in humans, rendering *Drosophila* a suitable and efficient model system to better understand the molecular nature of this pathway. In the present study, we review the current understanding of the molecular mechanism and clinical impact of the Hippo pathway. Current studies have demonstrated that a variety of deregulated molecules can alter Hippo signaling, leading to the constitutive activation of the transcriptional activator YAP or its paralog TAZ. Additionally, the Hippo pathway integrates inputs from a number of growth signaling pathways, positioning the Hippo pathway in a central role in the regulation of tissue size. Importantly, deregulated Hippo signaling is frequently observed in human cancers. YAP is commonly activated in a number of *in vitro* and *in vivo* models of tumorigenesis, as well as a number of human cancers. The common activation of YAP in many different tumor types provides an attractive target for potential therapeutic intervention.

## Introduction

Cancer is the second leading cause of death in the United States, with more than 1.6 million new cases estimated in 2014 [1]. Our ability to identify and understand the molecular lesions that lead to human tumor development is essential to improving the diagnosis and treatment of cancer and, ultimately, the long-term survival of cancer patients. However, the molecular signal transduction pathways that drive tumor development are still not entirely understood. These pathways have the potential to provide both diagnostic markers and novel therapeutic targets with which to treat cancer. The appreciation of deregulated signal transduction pathways continues to contribute to personalized medicine in cancer patients, where the identification of the specific molecular alterations associated with each patient’s tumor helps to optimize treatment (reviewed by Huang et al. [2]).

The Hippo signal transduction pathway is an essential regulator of organ size during developmental growth. Mutations in this pathway, first discovered in *Drosophila melanogaster*, have consistently demonstrated that dysfunctional Hippo pathway signaling leads to dramatic tissue overgrowth. In the present study, we review the basic components of the Hippo pathway, the evidence that deregulation of this pathway leads to well-conserved tumor-like phenotypes in model systems, and the role of Hippo signaling in cancer and human disease. A complete understanding of how the Hippo pathway contributes to disease in humans has the potential to lead to diagnostic improvements and novel therapeutics, highlighting the experimental relevancy of model systems to human disease research [3]. Overall, we find that despite a large array of upstream mechanisms that feed into the Hippo pathway, the evidence suggests that all mechanisms of deregulation result in the common activation of the transcription factors Yes-associated protein (YAP) and transcriptional co-activator with PDZ-binding motif (TAZ). This shared downstream output of the deregulated Hippo pathway provides attractive therapeutic targets that may have the potential to treat patients that exhibit a variety of molecular alterations that feed into the Hippo pathway.

## Review

### The molecular nature of the Hippo signaling pathway

**Figure 1:**
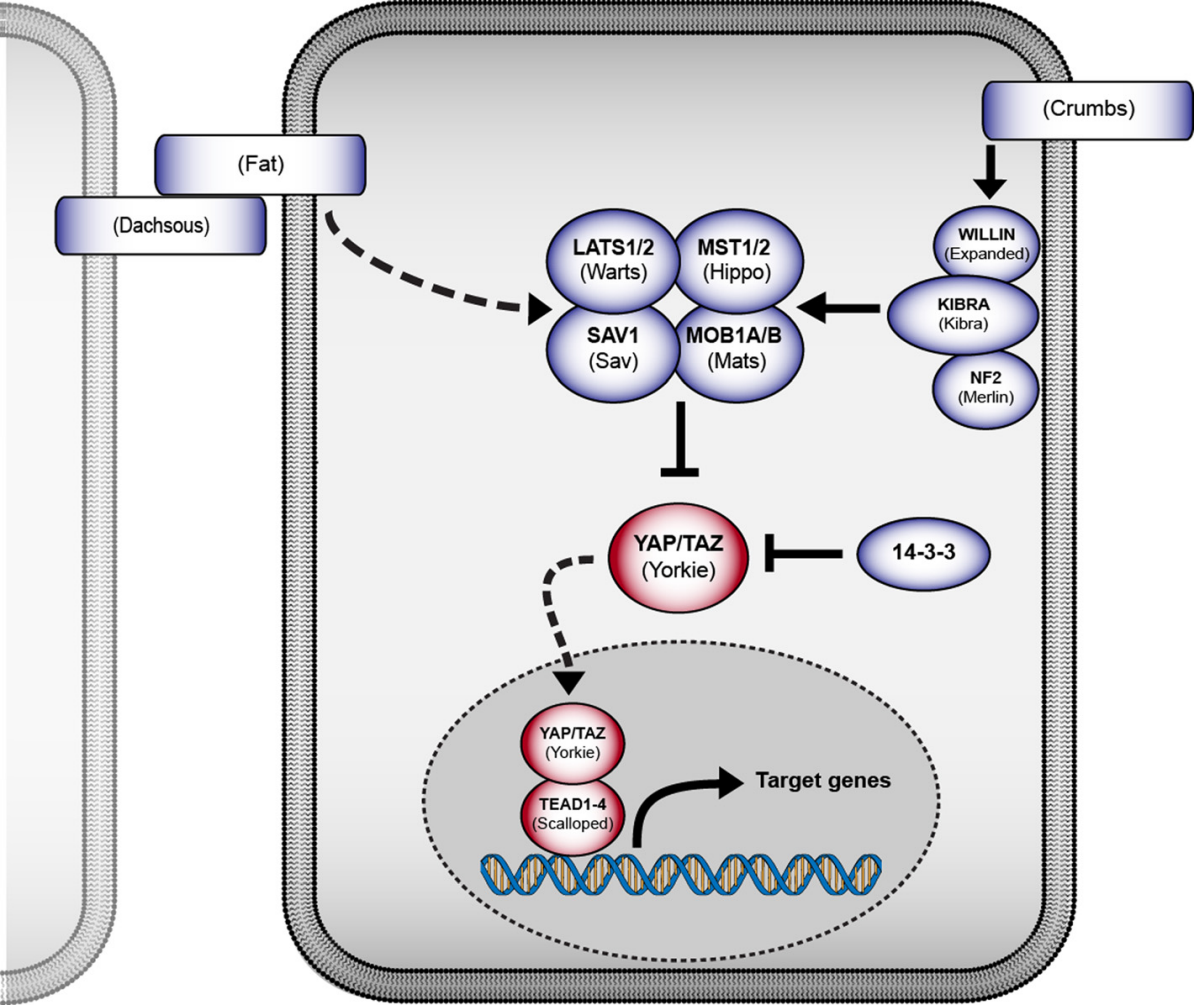
**Schematic of the core Hippo signaling pathway in *Drosophila *and humans.** Human molecules are in bold, *Drosophila* molecules in parentheses. Regulators, which act to restrict YAP/TAZ/Yki activation, are blue, and downstream transcriptional effectors are red.

The basic molecular architecture of the Hippo signaling pathway is conserved between *Drosophila* and humans. Notably, there is a greater level of molecular complexity and redundancy observed in the human Hippo pathway, often with multiple mammalian homologs for a single *Drosophila* protein. In humans, MST1/2 (Hippo in *Drosophila*) serve as upstream kinases that function to phosphorylate Lats1/2 (Warts in *Drosophila*) (Figure 1) (reviewed by Hilman and Gat [4,5]). Lats1/2 and MST1/2 are brought into close spatial proximity by the scaffold protein SAV1 (Salvador in *Drosophila*). MOB1A and MOB1B (Mats in *Drosophila*) function to enhance the kinase activity of Lats1/2 (reviewed by Hariharan [6,7]). Once activated, Lats1/2 continues the kinase cascade by phosphorylating the transcriptional co-activator YAP and its vertebrate-specific paralog TAZ (Yorkie (Yki) in *Drosophila*). Phosphorylated YAP (serine 127) is sequestered in the cytoplasm via a 14-3-3 protein family member, preventing it from entering the nucleus [8]. When the MST1/2 kinase cascade is inactivated, the YAP protein remains unphosphorylated, translocates into the nucleus, and activates transcription of target genes [9], and reviewed by Pobbati and Hong [10]. YAP does not have DNA binding capabilities and requires a binding partner to activate gene transcription. The most well studied transcriptional partners of YAP are the TEAD transcription factors (TEAD 1-4) (Scalloped in *Drosophila*). The TEAD proteins are unable to activate gene transcription on their own; however, when YAP is present in the nucleus they function together to promote transcription of a number of pro-proliferative and anti-apoptotic genes. Though the relationship between YAP/TEAD is the best understood, YAP has also been demonstrated to associate with additional DNA binding proteins during transcriptional activation, including SMAD family transcription factors [11]. In addition to the robust conservation of core molecular components of the pathway, the transcriptional program of an activated YAP/Yki, which promotes cellular proliferation and survival, is also conserved [12-14]. Though flies and humans achieve this transcriptional program through the activation of different genes, the cellular outcome of the transcriptional profile remains conserved*.*

Though Hippo signaling converges on a singular output of YAP/Yorkie translocation to the nucleus, a variety of upstream signals converge on the core MST1/2 kinase cassette to input into the Hippo pathway. For instance, three proteins—KIBRA (Kibra in *Drosophila)*, WILLIN (Expanded in *Drosophila*), and NF2 (Merlin in *Drosophila*) —have been shown to aid in localization of the core Hippo proteins to junctional complexes, which is critical for their activation within the cell (reviewed by Grusche et al. [15]) [16-19]. In *Drosophila* the most well understood upstream regulators of Hippo signaling are those related to cell polarity and cell–cell contact. Fat and Dachsous are atypical cadherins that signal as negative upstream regulators of the Hippo pathway (reviewed by Grusche et al. [15]). The expression of Fat and Dachsous integrates information encoded by morphogen gradients (Hedgehog, Wingless) to provide a molecular mechanism regulating organ size through Hippo activity [20]. Crumbs, a protein involved in maintaining apical-basal polarity, was identified as an upstream regulator of Hippo signaling which acts through the localization of Expanded [21-23]. Although homologs of Crumbs, Fat, and Dachsous have identified human homologs, their specific roles in vertebrate Hippo signaling are not as well understood. For instance, humans have four *FAT* genes (*FAT1-4*), but their role in Hippo signaling is still unclear, reviewed by Sadeqzadehet et al. [24]. In zebrafish, fat1 has been shown to bind scribble to influence Hippo signaling [25]. However, FAT4 is arguably the closest structural and functional mammalian ortholog of *Drosophila* Fat [24,26]. Nonetheless, recent findings have shown that a conditional knockout of FAT4 in mouse livers failed to result in liver overgrowth or tumorigenesis, obscuring the role of FAT4 in Hippo-mediated mammalian overgrowth [27].

Recent proteomic studies in both *Drosophila* and humans have found hundreds of potential novel Hippo pathway interactors and regulators; with future mechanistic studies, these interactors could prove to be important regulators or downstream effectors of the pathway [28-31].

### Cellular inputs that alter hippo signaling

**Figure 2:**
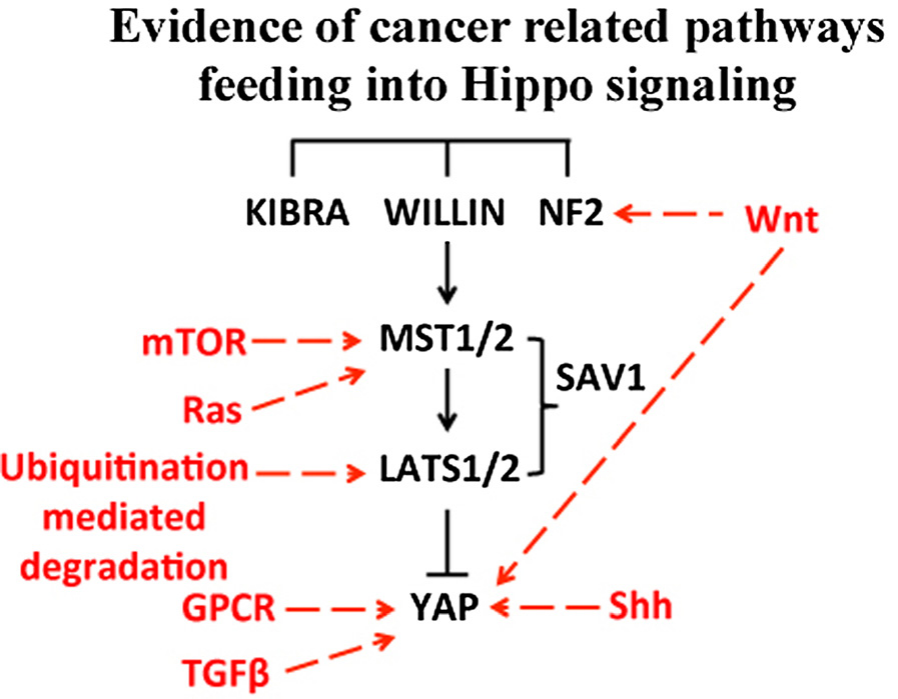
**Schematic of cancer related pathways and processes that input into the Hippo pathway.** Core mammalian Hippo pathway components are in black, and additional cancer related molecular pathways are in red.

The core of the Hippo pathway, with several kinases negatively regulating YAP, is augmented by a number of different pathways that provide input into this core cassette*,* including cellular polarity, cell-to-cell contact, and G-protein coupled receptor (GPCR) signaling. Different GPCR signaling pathways have been shown to both activate and inhibit YAP depending on the specific G-protein activated [32]. In addition to the alterations on the core Hippo pathway, a number of other molecular pathways commonly altered in human carcinogenesis have the ability to cross talk with the Hippo pathway (reviewed by Irvine [33]). Wnt signaling is activated through cytoplasmic β-catenin. A transcriptional target of β-catenin is CD44, which has the ability to activate of NF2, providing a mechanism in which Wnt signaling can activate the Hippo pathway [34]. Wnt pathway mutations have been shown to increase the nuclear localization of YAP, and YAP has been shown to interact physically with β-catenin [34,35]. TGF-β signaling activates Smad1, which has the ability recruit YAP to the nucleus [11]. Moreover, Smad1/Mad is a direct binding partner for YAP/Yki in both *Drosophila* and humans [11,36]. mTOR demonstrates crosstalk with the Hippo pathway, with the ability to both activate and be activated by Hippo signaling [37]. Mutations in the gene *patched,* the transmembrane receptor for the Hedgehog signaling pathway, have been demonstrated to activate Hippo signaling in both *Drosophila melanogaster* models and human tumors [38,39]. The Ras pathway’s RASSF proteins (dRASSF in *Drosophila*), a family of proteins that have been shown to associate with Ras, serve as negative regulators of the Hippo pathway [15,40]. Hippo signaling is also regulated in a post-translational manner through the ubiquitination of Lats1 by the E3 ligase ITCH. Alterations in the regulation of this post-translational modification can deregulate the pathway [41]. These studies point to a model, which features a centralized role for the Hippo pathway in integrating growth signals from a number of pathways that regulate tissue/organ size (Figure 2). Importantly, the widespread deregulation of many of these pathways in human cancer leads to the secondary inactivation of Hippo signaling, ultimately contributing to tumorigenesis (reviewed by Yu and Guan [42] and reviewed by Irvine [33]).

### Deregulation of hippo pathway alters several cancer related cellular processes

The genetic screens that led to the discovery of Hippo pathway factors in *Drosophila* relied on single recessive mutations to drive overgrowth phenotypes (reviewed by Hariharan and Bilder [43]). Some of the most dramatic mutant overgrowth phenotypes identified in these screens were mutations in negative regulators of the Hippo pathway (Hpo, Sav, Wts, Crbs, Ex), whose loss simultaneously altered several cellular processes associated with tumorigenesis including increased cellular proliferation and prevention of apoptosis [6]. Likewise, in humans, deregulation of the Hippo pathway leads to a number of cellular processes associated with cancer progression including increased cellular proliferation, inhibition of apoptosis, and the deregulation of cellular differentiation.

#### ***Increased cellular proliferation***

Mutations in Hippo pathway components in *Drosophila* consistently result in an increase in cell proliferation driven by excess Yorkie in the nucleus (reviewed by Pan [44]). Mouse models that have tissue specific activation of YAP demonstrate an increase in overall organ size, which is associated with an increase in cellular proliferation [45]. This YAP driven increase in proliferation occurs in a number of different developing murine tissues, suggesting a generalized role of YAP to promote cellular proliferation in mammalian cells [46,47]. In addition to YAP over-expression, genetic loss of *MST1/*2 has also been demonstrated to result in over-proliferation [48]. Human cell culture models further support a mechanism in which YAP or TEAD over-expression leads to an increase in the proliferation capability of a cell [14,49].

#### ***Inhibition of apoptosis***

An increase in the levels of DIAP1 protein, a key inhibitor of cell death in *Drosophila*, has been commonly observed in mutants that inactivate Hippo signaling [13,50,51]. In mouse models, MST1/2 have been demonstrated to be pro-apoptotic and their loss confers a resistance to apoptosis concurrent with YAP activation [46,52]. In human cell culture, activated YAP has been associated with an increase in survival proteins such as Survivin and IAP1 (reviewed by Dong et al. [53,54]). Interestingly, YAP has also been identified as a binding partner of the pro-apoptotic gene *p73*, suggesting that in certain cellular contexts, YAP may be pro-apoptotic [54,55].

#### ***Deregulation of cellular differentiation***

Hippo signaling also plays a role in cellular differentiation. MST1/2 are essential components of differentiation, and inactivation of Hippo signaling leads to the induction pluripotent stem cells [56]. In mouse cell culture models, YAP expression is associated with the maintenance of embryonic stem cells, and the reduction of nuclear YAP corresponds with differentiation [42,57]. Although the role of cancer-stem cells is controversial and not fully understood (reviewed by Brennan and Matsui [58]), deregulation of the Hippo pathway has the potential to induce stem cell–like properties such as increased proliferative capacity [59]. Therefore, the maintenance of an un-differentiated state in cells in which the Hippo pathway has been deregulated may further contribute to human cancer development.

### Hippo pathway signaling suppresses cancerous phenotypes in cell culture

Given the initial discovery in *Drosophila* that Hippo signaling suppresses tissue growth, it is not surprising that Hippo pathway components are implicated in the tumorigenic transformation of mammalian cells in culture. Indeed, *in vitro* work shows that both gain and loss of Hippo signaling components in a wide range of both non-cancer and cancer cell lines can enhance or suppress cancerous phenotypes [49,60-72]. Consistent for a central role of YAP/TAZ in the transforming properties of the Hippo pathway, the over-expression of YAP or TAZ in cell culture leads to transforming phenotypes including anchorage-independent growth, epithelial to mesenchymal transition, growth-factor independent proliferation, inhibition of apoptosis, resistance to chemotherapeutics, faster cell migration, tumor-initiation properties, invasion, and tumor formation in xenograft models [49,60-66]. Concordantly, removal of YAP or TAZ suppresses cancerous phenotypes in cancer cell lines [60-62,65-70]. For example, siRNA knockdown of YAP reduces cellular proliferation, induces apoptosis, and inhibits anchorage-independent growth in pancreatic cell lines [71]. Likewise, a decrease in YAP reduces the proliferative capability of breast cancer cells in culture [73]. Collaboratively knocking down both YAP and TAZ significantly reduces the ability of colon cancer cells to proliferate, metastasize, and invade [74].

Antagonistic to YAP and TAZ, an increase in the activity of the Hippo core kinase cassette restricts cancerous phenotypes in cell culture. For example, overexpression of Mst1 in non-small-cell lung cancer (NSCLC) cells inhibits cellular proliferation and survival through YAP phosphorylation [75]. A clear cell renal cell carcinoma (ccRCC) cell line with homozygous loss of *SAV1* has reduced colony-forming capacity when SAV1 is expressed exogenously; additionally, non-tumorigenic renal cells become more proliferative upon SAV1 knockdown [76].

### Hippo pathway components contribute to tumorigenesis in mouse models

Research extending from cell culture into mouse models has highlighted the ability of Hippo signaling components to drive mammalian cancer development. Overexpression of YAP in the mouse liver results in hepatomegaly, followed by tumorigenesis [46]. Furthermore, YAP expression contributes to tumor progression in mouse liver and lungs [69,77]. Loss of YAP suppresses oncogene-induced tumor growth in mouse mammary glands [78]. Lats1 null mice develop soft-tissue sarcomas and ovarian stromal cell tumors [79]. Mouse livers missing Mst1 and Mst2, or SAV1, have elevated YAP activity leading to hepatomegaly and hepatocellular carcinoma (HCC) [22,80-82]. Additionally, mice that are null for Mst1 and Mst2 in the intestinal epithelium develop adenomas in the distal colon and possess an expanded undifferentiated stem cell compartment throughout their intestines [83]. Expression of the Mst1 gene in a NSCLC cell line suppresses tumor growth in a mouse xenograft model [75]. Together, these data support a conserved tumor suppressor role for Hippo signaling in mouse models of human tumorigenesis.

### Molecular alterations of hippo pathway components in human cancer

In addition to work done in cell culture and model systems, a number of studies have shown that YAP/TAZ are activated in a wide range of human cancers, bolstering the evidence that deregulated Hippo signaling contributes to carcinogenesis. Using immunohistochemistry, YAP has been found to be either strongly expressed or highly localized to the nucleus (where it is active in gene transcription) in human cancers compared with normal tissue. Increased expression and/or nuclear accumulation of YAP has been reported in a wide array of human cancers including HCC, prostate cancer, colorectal carcinoma (CRC), NSCLC, ovarian cancer, ccRCC, pancreatic carcinoma, esophageal squamous cell carcinoma, urothelial carcinoma of the bladder, and skin basal cell carcinoma (Additional file 1: Table S1) [34,61-63,67,71,72,76,83-87]. Notably, expression or nuclear localization of YAP is associated with poorer tumor differentiation and higher-grade tumors [76,86]. Concordantly, TAZ is overexpressed in high-grade breast cancers, CRCs, and tongue squamous cell carcinomas [66,74,88]. Negative regulators of YAP/TAZ signaling including Mst1/2, Lats1/2, NF2, Mob1*,* and SAV1 exhibit loss of expression in human tumors (reviewed by Zhao et al. [45] and Harvey et al. [89]). For example, a majority of human HCCs have inactivated Mst1, and approximately 30% have reduced YAP1 phosphorylation at the inhibitory S127 site [80].

Given the evidence for YAP/TAZ activation, there is a surprising lack of documented genetic mutations in core Hippo components in cancer [89]. As sequencing studies continue, a greater number of mutations in Hippo pathway components may be revealed in different subsets of tumors. Nonetheless, the current dearth of *bona fide* cancerous mutations in the Hippo pathway is striking. A number of factors could help to explain this surprise. Firstly, the mammalian Hippo pathway has redundant upstream negative regulators of YAP/TAZ (*i.e.* Mst1/2 and Lats 1/2) such that multiple mutations would be required to inactivate the pathway in a tumor. Secondly, other mechanisms of Hippo pathway alteration have been discovered in various human cancers, including copy-number changes, translocations, and epigenetic silencing, which could explain the observed YAP/TAZ activation without associated mutations in Hippo pathway genes. Thirdly, the activation of YAP/TAZ could be the result of other growth regulating pathways (*i.e.* Ras, TGF-β, and Wnt) exerting suppressive pressure on Hippo signaling, such that deregulation of those pathways leads indirectly to the activation of YAP/TAZ [42,89-95].

Nevertheless, one prominent example of genetic mutation affecting the Hippo pathway is Neurofibromatosis 2, a dominantly inherited familial syndrome caused by mutations in the *NF2* gene. This syndrome has an incidence of approximately 1:25,000 and a prevalence of approximately 1:80,000. Notably, patients with Neurofibromatosis 2 are predisposed to developing tumors of the nervous system, including schwannoma, meningioma, ependymomas, and astrocytomas [90]. Nearly all patients acquire bilateral vestibular schwannomas by age 30 [91]. In addition to germline *NF2* mutations, somatic mutations have been found in sporadic human tumors, primarily those that originate in the nervous system [89]. Mutations of *NF2* disrupt Merlin, an upstream regulator of the Hippo pathway, which may explain the tumor predisposition seen in patients. Concordantly, NF2 acts as a tumor suppressor through Hippo signaling in mice, where conditional knockout of NF2 in mice liver resulted in HCC [92]. Furthermore, the tumor cell proliferation in human schwannomas has recently been linked to a gene expression network controlled by YAP [93].

Additional examples of somatic mutations in Hippo pathway components are rare. In the Catalogue of Somatic Mutation in Cancer (COSMIC) database, approximately 1% to 2% of the more than 5,000 unique human cancer samples contain nonsynonymous mutations in *Lats1* or *Lats2*[94,96]. Recently, these *Lats1/2* cancer mutations were shown to disrupt a number of *Lats1/2* functions, including kinase activity, suppression of YAP activity, and tissue growth properties. These data, along with the identified mutations, serve to bolster the evidence for *Lats1/2* as tumor suppressor genes in a small subset of cancers [96]. Homozygous deletions of the *FAT1* gene, a putative upstream negative regulator of Hippo signaling, were discovered in 23% of oral cancer cell lines and 80% of primary oral cancers [95]. However, given the disproportionately large size of the FAT genes, it is still unclear if FAT gene mutations are ‘passenger’ or ‘driver’ mutations in cancer.

In addition to genetic mutations, the Hippo pathway is disrupted via other mechanisms in cancer, including copy-number changes, loss of heterozygosity, and epigenetic silencing (Additional file 1: Table S1). In mice with inactivation of tumor suppressor genes *BRCA1* and *p53*, an amplicon containing only the *YAP* gene was isolated from mammary tumors [49]. However, overexpression of YAP alone does not promote oncogenic growth in mouse mammary glands, suggesting that YAP amplification requires these cooperating lesions to transform cells [78]. A similar amplicon (9qA1) containing the *YAP* gene was found at high frequency in murine tumors derived from Myc-expressing cells. These amplicons are syntenic to a larger human locus (11q22) that is amplified in 5% to 10% of tumor types, including lung, ovarian, esophageal, and liver carcinomas [77]. The *TAZ* locus is amplified in 27 of 313 breast tumors, and *TAZ* mRNA expression was specifically increased in the tumors with the amplification [66]. The *TAZ* locus has also been implicated in a disease-defining chromosomal translocation in a rare vascular sarcoma termed epithelioid hemangioendothelioma. Endothelial cells highly expressing the product of this translocation, which fuses TAZ to the calmodulin-binding transcription activator 1 (CAMTA1), are associated with this cancer, although the oncogenic mechanism of this fusion protein has yet to be elucidated [97,98]. Studies have also shown copy number loss of *SAV1* in high-grade ccRCC [76], and loss of heterozygosity at the *FAT1* locus has been reported in primary glial tumors [99].

Epigenetic silencing affects the expression of several members of the Hippo pathway in a variety of human cancers. For example, more than half of human breast tumors have hypermethylation at either the *Lats1* or *Lats2* CpG island, resulting in lower expression of *Lats1/2* mRNA and an association with more aggressive tumors [100]. Similar results were detected in human astrocytomas and CRC [101,102]. Analogously, *Mst1* and *Mst2* are down-regulated in human soft tissue sarcoma due to CpG island hypermethylation [103]. Upstream Hippo inputs have also been found to have hypermethylated promoters with reduced gene expression, including *FAT4* in breast cancer and *KIBRA* and *FAT1* in B-cell acute lymphocytic leukemia (B-ALL) [104,105].

### YAP/TAZ activation is a prognostic indicator in cancer patients

Expression or nuclear localization of both YAP and TAZ has been associated with poor prognostic indicators and shorter survival times for patients with a wide range of human cancers. Specifically, YAP activation is associated with reduced patient survival in CRC, NSCLC, HCC, ovarian cancer, esophageal squamous cell carcinoma, and urothelial carcinoma of the bladder [60-63,74,86,87]. Recently, a *YAP* gene expression signature was validated as an independent predictor of prognosis in human ovarian cancer patients, and there was a significant association between YAP expression and tumor sensitivity to chemotherapeutic taxanes [106]. Similarly, TAZ expression has been shown to correlate with reduced survival in CRC, tongue squamous cell carcinoma, and recently breast cancer [65,68,74,88]. CRC patients with overexpression of both YAP and TAZ have worse outcomes than those who have either one alone [74], and a YAP/TAZ gene expression signature was significantly associated with worse overall survival and more frequent metastasis in lung adenocarcinoma patients [69]. Future clinical application of YAP/TAZ testing may improve cancer prognosis and treatment selection.

### Hippo pathway and non-cancer disease processes

In addition to its role in carcinogenesis, the Hippo pathway has been implicated in Sjogren’s syndrome, a chronic autoimmune disorder resulting in the destruction of the salivary and lacrimal glands. In mice, Hippo signaling is required for normal salivary gland development, and salivary glands from a mouse model of Sjogren’s syndrome phenocopy glands with Lats2 inhibition. Importantly, salivary glands from human Sjogren’s patients exhibit nuclear TAZ staining and upregulation of TAZ transcriptional targets [107].

The Hippo pathway is also implicated in tissue regeneration, including a requirement for YAP in the recovery of damaged mouse intestine [108]. Recently, YAP and TAZ have been shown to be upregulated in mouse wounds, and knock down of YAP and TAZ delays wound closure [109]. Further insight into the regenerative properties of downstream Hippo signals could yield important therapies for tissue healing.

### Potential therapeutics targeting the hippo pathway

The Hippo pathway’s contribution to disease pathogenesis has sparked interest in the development of potential therapeutics that could target key effectors of the signaling cascade. No matter the mechanism of Hippo pathway inactivation, cancer cells frequently exhibit hyperactive YAP, suggesting that YAP is a central contributor to tumorigenesis. YAP’s ability to also function as a tumor suppressor in certain cellular contexts, where it can act in collaboration with p73 to promote apoptosis, complicates YAP-targeted cancer therapies (reviewed by Wang et al. [54,55,72]). Nonetheless, YAP serves as an attractive clinical target to treat tumors with Hippo pathway deregulation. Not surprisingly, early efforts to develop YAP-targeting therapeutics have begun. For instance, in a cell-based screen, the drug dobutamine, a β-adrenergic receptor antagonist, has been shown to recruit YAP to the cytosol and inhibit YAP-dependent gene transcription through a mechanism unrelated to core Hippo signaling [110]. In addition, a small molecule drug named verteporfin, used in the laser-activated ablation of blood vessels in macular degeneration, has been identified as an inhibitor of TEAD-YAP association and YAP-induced liver overgrowth [111]. Recently, verteporfin was shown to suppress growth in breast cancer cell lines; the cell lines with the most YAP expression were also the most sensitive to verteporfin [78]. A newly characterized tumor suppressor gene named *VGLL4* has been shown to inhibit the activity of the YAP-TEAD complex by competing with YAP for binding to TEADs via tandem Tondu domains [112,113]. Notably, a peptide that mimics the YAP-TEAD inhibitor activity of VGLL4 was shown to suppress gastric tumor growth *in vitro* and *in vivo*[113]. Together, these early advancements in targeting YAP have generated excitement over prospective new therapeutics to treat cancer and other disease.

## Conclusions

The Hippo pathway is a conserved signaling cascade that serves as a developmental regulator of organ size and, when deregulated, fuels carcinogenesis. Model systems, including *Drosophila*, mice, and cell culture, have provided insight into the molecular relationship of components within the Hippo pathway and their control of cell division, apoptosis, and ultimately, organ size. The Hippo pathway also integrates signals from a number of established growth pathways, including mTOR, Wnt, and Ras. Pertinent to human disease, the deregulation of Hippo pathway components promotes cancer development through multiple mechanisms including inactivating mutations in upstream regulators, epigenetic alterations, loss of heterozygosity, alterations in copy number, and deregulation of associated molecular pathways. Regardless of their mechanism, these alterations yield the same molecular end result—the expression of active, nuclear-localized YAP/TAZ. Targeting the constitutive activation of YAP/TAZ for therapeutic treatment may address a diverse spectrum of different molecular alterations funneling into a common transcriptional activator. This shared molecular outcome presents both YAP and TAZ as attractive diagnostic and therapeutic markers for an array of different human cancers.

## Abbreviations

(GPCR): G-Protein coupled receptor; (NSCLC): Non-small-cell lung cancer; (ccRCC): Clear cell renal cell carcinoma; (HCC): Hepatocellular carcinoma; (CRC): Colorectal carcinoma; (COSMIC): Catalogue of somatic mutation in Cancer; (CAMTA1): Calmodulin-binding transcription activator 1; (B-ALL): B-cell acute lymphocytic leukemia; (Ub): Ubiquitination.

## Competing interests

The authors declare that they have none no competing interests.

## Author’s contributions

DAB wrote and edited the manuscript and created Figure 1 and Additional file 1: Table S1. JDK wrote and edited the manuscript and created Figure 2. All authors read and approved the final manuscript.

## Supplementary Material

Additional file 1: Table S1 Evidence of Hippo pathway deregulation in human cancer. Activating events are in green and inactivating events are in red. The asterisks indicate that this mutation is found in only a small subset of cancers (1-2%). Click here for file
